# Iodine concentration in the lung parenchyma in relation to different inspiratory depths during CT pulmonary angiography

**DOI:** 10.1093/bjr/tqaf008

**Published:** 2025-01-16

**Authors:** Koichiro Yasaka, Hiroyuki Saigusa, Osamu Abe

**Affiliations:** Department of Radiology, Graduate School of Medicine, The University of Tokyo, Tokyo 113-8655, Japan; Department of Radiology, Graduate School of Medicine, The University of Tokyo, Tokyo 113-8655, Japan; Department of Radiology, Graduate School of Medicine, The University of Tokyo, Tokyo 113-8655, Japan

**Keywords:** contrast media, lung, multidetector CT, CT angiography

## Abstract

**Objectives:**

This study aimed to investigate the impact of changing inspiratory depth from end- to mid-inspiratory level on the iodine concentration in the lung parenchyma and main pulmonary artery in dual-energy CT pulmonary angiography.

**Methods:**

This retrospective study included patients who underwent dual-energy CT pulmonary angiography from July 2020 to June 2023. Patients were instructed to hold their breath at end- and mid-inspiratory levels before and after January 2022, respectively. By placing regions of interest on the lung lobes and main pulmonary artery in the iodine map, their iodine concentration was recorded.

**Results:**

In end- and mid-inspiratory command, 173 (mean age: 63.4 ± 17.0 years; 68 males) and 179 (mean age: 65.1 ± 15.4 years; 62 males) patients, respectively, were included. The mean iodine concentrations of the right upper, right middle, right lower, left upper, and left lower lobes were 0.81/0.91, 0.67/0.74, 1.06/1.07, 0.85/0.95, and 1.07/1.11 mgI/mL, respectively, for the end-/mid-inspiratory level. The multivariable regression analysis revealed inspiratory depth as a significant factor for iodine concentration of the right upper, right middle, and left upper lobes. Main pulmonary artery iodine concentration in mid-inspiratory depth (13.21 mgI/mL) was higher than that in end-inspiratory depth (12.51 mgI/mL) (*P *=* *.129), and a statistically significant difference was observed in the patient group with a body weight of ≥70 kg (*P *=* *.015).

**Conclusions:**

Changing inspiratory depth from end- to mid-inspiratory level has a significant impact on the iodine concentration in the upper and right middle lobes in dual-energy CT pulmonary angiography.

**Advances in knowledge:**

Changing inspiratory depth from end- to mid-inspiratory level has significantly increased the iodine concentration in the right upper, right middle, and left upper lobes.

## Introduction

Pulmonary embolism is generally the third most common cause of death from cardiovascular disease, following heart attack and stroke.^1^ An established modality in detecting pulmonary embolism includes CT pulmonary angiography.^2^ Pulmonary embolism CT imaging has evolved since dual-energy CT technology emerged. The iodine map is considered the most valuable dual-energy CT image, while several applications of dual-energy CT exist, including iodine map, monochromatic imaging, and virtual noncontrast imaging.^3^ The iodine map reveals peripheral defects in patients with pulmonary embolisms.^4^ Weidman et al.^5^ reported that pulmonary embolism was detected in 12.8% of CT pulmonary angiographies at initial review, and additional pulmonary embolisms are found in 2.3% after the iodine map review. However, transient interruption of contrast remains a disadvantage in CT pulmonary angiography^6^ in which pulmonary artery CT attenuation is decreased due to the increased reflux blood volume from the inferior vena cava during inspiration at CT scan,^7^ in some patients.

Some strategies manage the decreased image quality of CT pulmonary angiography, such as low kVp scan^8^ and highly concentrated iodinated contrast agents combined with high injection flow rates.^9^ However, the former method suffers from increased image noise, and the latter is associated with increased artefacts. Changing inspiratory depth is another strategy for managing problems with transient interruption of contrast. Reportedly, changing the inspiratory depth from end- to mid-inspiratory level helped reduce the incidence of transient interruption of contrast from 6.5% to 1.9%.^10^ The iodine concentration in the lung parenchyma is expected to be increased at the mid-inspiratory level. However, to our knowledge, no reports have investigated the association of inspiratory depth and iodine concentration in the lung parenchyma and pulmonary artery in dual-energy CT pulmonary angiography.

This study aimed to assess the impact of changing the inspiratory depth from end- to mid-inspiratory level on the iodine concentration in the lung parenchyma and the pulmonary artery in dual-energy CT pulmonary angiography.

## Materials and methods

Our Institutional Review Board, which waived the requirement for obtaining written informed consent from patients due to its retrospective nature, approved this study.

### Patients

**Figure 1.**
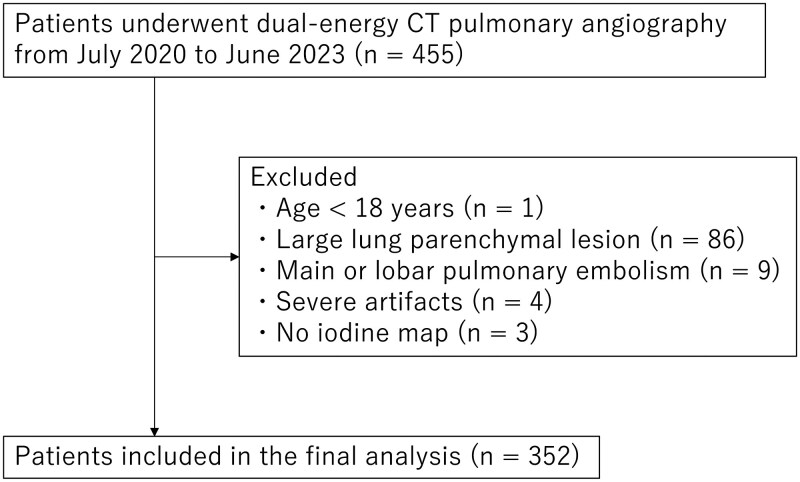
Flowchart of the patient inclusion and exclusion process.

We searched patients who underwent dual-energy CT pulmonary angiography with a CT scanner from July 2020 to June 2023. The following patients were excluded from 455 patients who met the inclusion criteria: age <18 years (*n* = 1); with lung parenchymal lesions, such as atelectasis, consolidation, and emphysema, occupying whole lobe (*n* = 86); with pulmonary embolism larger than lobe branch (*n* = 9); with severe artefacts (*n* = 4); and without iodine map (*n* = 3). Hence, the final analysis included 352 patients. Figure 1 shows the patient inclusion and exclusion process.

### CT imaging

All patients underwent CT pulmonary angiography with a single CT scanner (Revolution CT [GE Healthcare, WI, USA]). Our hospital changed the inspiratory depth from end-inspiratory to mid-inspiratory level (instructed to hold the breath at approximately half of maximal inspiratory volume) in January 2022 to reduce transient interruption of contrast. CT scan was performed with dual-energy mode (fast kVp switching with 80 kVp and 140 kVp). Other CT scanning parameters were tube current, auto mA with noise index set at 11.4; gantry rotation time at 0.5 s; pitch at 0.992. Parameters for iodine map reconstruction were slice thickness/interval at 1.25 mm/1 mm and field of view at 350 mm. Contrast material (600 mgI/kg) was injected from the right or left antecubital vein within 30 s using an automatic power injector. The scan was started 20 s after starting the contrast injection. The iodine concentration of the contrast agent was determined based on the body weight: 320, 350, and 370 mgI/mL for <48, 49–63, and >64 kg, respectively, in principle. This study used the following contrast agents: iopamidol (Bayer) (*n* = 122), optiray (Guerbet) (*n* = 48), omnipaque (GE Healthcare) (*n* = 166), and iomeron (Bracco) (*n* = 16).

### CT image evaluation

**Figure 2.**
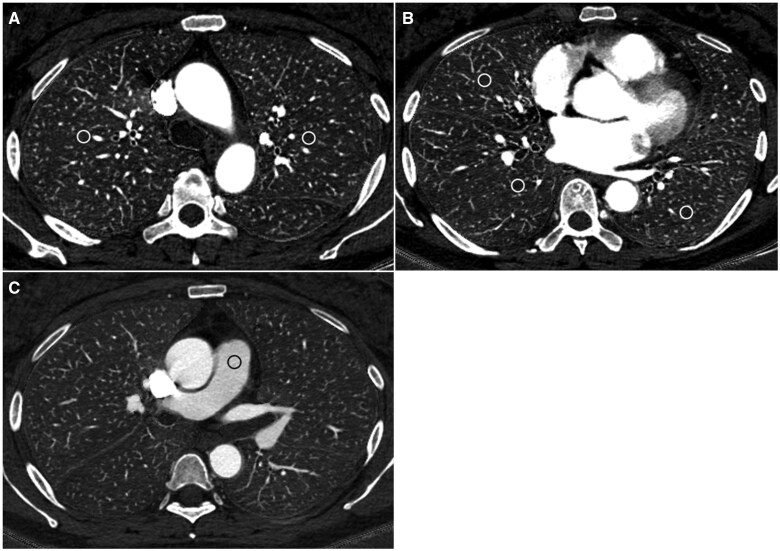
Regions of interest (white or black circles) were placed on the right upper and left upper (A), right middle, right lower, and left lower lobes (B), and main pulmonary artery (C).

A radiologist with imaging experience of 13 years placed regions of interest with diameters of 8–10 mm on the right upper, middle, and lower lobes; left upper and lower lobes; and the main pulmonary artery in an iodine map (Figure 2) with reference to a conventional CT pulmonary angiography image. The following were avoided when placing regions of interest on the lung parenchyma: subpleural region, large vessels, lung lesions, regions with severe artefacts, and territories where pulmonary embolism is present in the proximal pulmonary artery. The mean iodine concentration for each of the regions of interest was recorded after placing them.

Measurements were repeated for all the patients on different days to enhance their reproducibility after completing the above evaluations. The iodine concentration for each structure between the first and second measurements was averaged, and the averaged values were used for the following analysis.

### Statistical analysis

Statistical analysis was performed with R version 4.1.2 (The R Foundation for Statistical Computing). The iodine concentration of the contrast agent was dichotomized to ≤350 mgI/mL and >350 mgI/mL in the following evaluations. Fisher’s exact test and Student’s *t*-test were performed to compare nominal and continuous variables between end- vs mid-inspiratory command, respectively. *P*-value of <.050 was considered to indicate statistically significant difference for these analyses.

Lung parenchymal iodine concentrations were compared between each lobe with paired *t*-test. The Bonferroni correction was performed, and *P*-values <.005 (= .050/10) was considered to indicate a statistically significant difference for this analysis because of multiple comparisons (10 comparisons).

Pearson’s correlation coefficient was calculated to assess the relationship between factors of continuous variables and parenchymal iodine concentration. Parenchymal iodine concentration was compared with the Student’s *t*-test for factors of nominal variables. Multivariable regression analysis with stepwise selection based on Akaike’s information criterion was performed to find significant factors that affect the parenchymal iodine concentration. This analysis used factors with *P*-values of <.100 in the univariable analysis as input data.

Intraclass correlation coefficient (1, 2) was used to assess agreement for the averaged iodine concentration values.

## Results

### Patients

**Figure 3.**
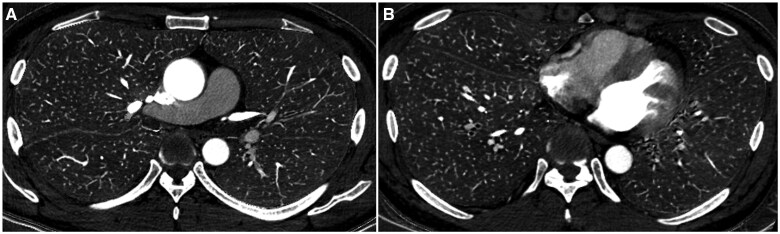
Iodine maps (upper zone [A] and lower zone [B]) of a 48-year-old male patient with a body weight of 71 kg in whom transient interruption of contrast was seen. This patient underwent CT pulmonary angiography at the end-inspiratory level. A contrast agent with an iodine concentration of 370 mgI/mL was injected from the right antecubital vein. The iodine concentration of the right upper, right middle, right lower, left upper and left lower lobes, and main pulmonary artery was 0.69, 0.52, 0.45, 0.52, 0.58, and 3.9 mgI/mL, respectively.

**Figure 4.**
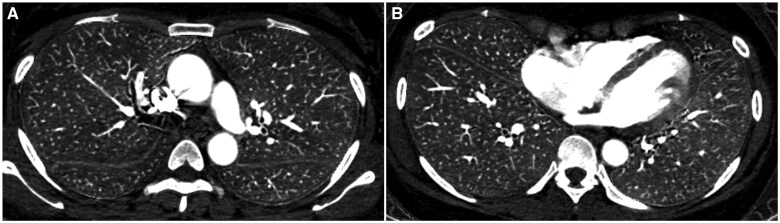
Iodine maps (upper zone [A] and lower zone [B]) of a 42-year-old female patient with a body weight of 64 kg. This patient underwent CT pulmonary angiography at the mid-inspiratory level. A contrast agent with an iodine concentration of 370 mgI/mL was injected from the right antecubital vein. The iodine concentration of the right upper, right middle, right lower, left upper and left lower lobes, and main pulmonary artery was 1.12, 1.09, 1.16, 1.20, 1.67, and 15.9 mgI/mL, respectively.

Table 1 summarizes the patient background information. End- and mid-inspiratory commands included 173 (mean age: 63.4 ± 17.0 years; 68 males) and 179 (mean age: 65.1 ± 15.4 years; 62 males) patients. No statistically significant difference in patient background was found between end- and mid-inspiratory commands (*P *≥* *.245). Figure 3 shows a representative iodine map of a patient performed at end-inspiratory level in whom transient interruption of contrast was seen. Figure 4 shows an iodine map of a patient performed at mid-inspiratory level.

**Table 1.** Patient background information.

	End-inspiratory level	Mid-inspiratory level	*P*-values
Number of patients	173	179	N/A
Age (years)	63.4 ± 17.0	65.1 ± 15.4	.326
Body weight (kg)	58.2 ± 13.1	58.3 ± 16.2	.935
Sex			.379
Male	68	62	
Female	105	117	
Iodine contrast agent			.497
≤350 mgI/mL	112	123	
>350 mgI/mL	61	56	
Side of injection			.245
Right	131	145	
Left	42	34	

Comparisons were performed with Fisher’s exact test and Student’s *t*-test for nominal and continuous variables, respectively. Mean ± standard deviation is shown for continuous variables.

Abbreviation: N/A: not applicable.

### Iodine concentration of each lung lobe

Table 2 shows the iodine concentration of each lung lobe. Iodine concentrations of the right upper, middle, lower, left upper, and lower lobes were 0.86, 0.70, 1.06, 0.90, and 1.09 mgI/mL, respectively. Statistically significant differences in iodine concentration were found between each lobe except for between the right lower lobe vs the left lower lobe (*P *=* *.276).

**Table 2.** Iodine concentration of each lung lobe.

	Values (mgI/mL)	*P*-values			
		Vs right middle lobe	Vs right lower lobe	Vs left upper lobe	Vs left lower lobe
Right upper lobe	0.86 ± 0.38	<.001*	<.001*	.002*	<.001*
Right middle lobe	0.70 ± 0.35	N/A	<.001*	<.001*	<.001*
Right lower lobe	1.06 ± 0.46	N/A	N/A	<.001*	.276
Left upper lobe	0.90 ± 0.42	N/A	N/A	N/A	<.001*
Left lower lobe	1.09 ± 0.48	N/A	N/A	N/A	N/A

Comparisons were performed with paired *t*-tests.

* Statistically significant difference.

Abbreviation: N/A: not applicable.

### Relation of factors and parenchymal iodine concentration: Univariable analysis

Tables 3 and 4 show the detailed results for the association of parenchymal iodine concentration vs factors with continuous and nominal variables, respectively. Only the inspiratory depth was a significant factor for iodine concentration of the right and left upper lobes (*P *≤* *.022). The iodine concentration of the right and left upper lobes was 0.81/0.91 and 0.85/0.95 mgI/mL, respectively, for end-/mid-inspiratory command. Sex (*P *=* *.002) and inspiratory depth (*P *=* *.0496) were significant factors for the right middle lobe. Iodine concentration of the right middle lobe was 0.63 and 0.75 mgI/mL for males and females, respectively, and 0.67 and 0.74 mgI/mL for end- and mid-inspiratory depth, respectively. There was no significant factor affecting iodine concentration for the right and left lower lobes.

**Table 3.** Pearson’s correlation coefficients for age and body weight vs parenchymal iodine concentration: Univariable analysis.

	Age		Body weight	
	Value	*P*-values	Value	*P*-values
Right upper lobe	0.067 (−0.038 to 0.170)	.212	0.057 (−0.047 to 0.161)	.283
Right middle lobe	−0.002 (−0.107 to 0.102)	.963	−0.094 (−0.196 to 0.011)	.079
Right lower lobe	0.078 (−0.027 to 0.181)	.144	−0.021 (−0.125 to 0.084)	.692
Left upper lobe	0.024 (−0.081 to 0.128)	.654	0.063 (−0.042 to 0.167)	.238
Left lower lobe	0.065 (−0.039 to 0.169)	.221	−0.059 (−0.163 to 0.046)	.267

Pearson’s correlation coefficient (with a 95% confidence interval) is shown.

**Table 4.** Comparisons of iodine concentration based on each factor: Univariable analysis.

	Right upper lobe	Right middle lobe	Right lower lobe	Left upper lobe	Left lower lobe
Sex					
Male	0.85 ± 0.43	0.63 ± 0.31	1.02 ± 0.48	0.86 ± 0.48	1.04 ± 0.48
Female	0.87 ± 0.36	0.75 ± 0.36	1.09 ± 0.45	0.93 ± 0.37	1.11 ± 0.47
*P*-values	.672	.002*	.183	.135	.204
Side of injection					
Right	0.85 ± 0.38	0.69 ± 0.34	1.05 ± 0.45	0.89 ± 0.40	1.07 ± 0.46
Left	0.88 ± 0.40	0.75 ± 0.38	1.13 ± 0.50	0.93 ± 0.46	1.15 ± 0.54
*P*-values	.623	.214	.147	.475	.168
Iodine concentration					
≤350 mgI/mL	0.85 ± 0.35	0.73 ± 0.34	1.07 ± 0.45	0.90 ± 0.38	1.09 ± 0.48
>350 mgI/mL	0.88 ± 0.45	0.66 ± 0.35	1.05 ± 0.48	0.91 ± 0.48	1.08 ± 0.46
*P*-values	.434	.093	.594	.947	.966
Inspiratory depth					
End-inspiratory	0.81 ± 0.37	0.67 ± 0.35	1.06 ± 0.44	0.85 ± 0.40	1.07 ± 0.51
Mid-inspiratory	0.91 ± 0.39	0.74 ± 0.34	1.07 ± 0.48	0.95 ± 0.43	1.11 ± 0.45
*P*-values	.011*	.0496*	.844	.022*	.448

The mean ± standard deviation of parenchymal iodine concentration (mgI/mL) for each category is shown. Comparisons between categories were performed with Student’s *t*-test.

* Statistically significant difference.

### Relationship of factors and parenchymal iodine concentration: Multivariable analysis

The inspiratory depth (estimate = 0.10, *P* = .011) was found to be a significant factor (intercept = 0.81) with the multivariable regression analysis for parenchymal iodine concentration of the right upper lobe. The inspiratory depth (estimate = 0.10, *P *=* *.022) was a significant factor for the parenchymal iodine concentration of the left upper lobe (intercept = 0.85). Inspiratory depth (estimate = 0.07, *P *=* *.066) and sex (estimate = 0.12, *P *=* *.002) remained factors (intercept = 0.71) for parenchymal iodine concentration of the right middle lobe. No significant factor affected the parenchymal iodine concentration of the lower lobes.

### Correlation of iodine concentration between the main pulmonary artery and lung parenchyma

The iodine concentration of the main pulmonary artery in mid-inspiratory command (13.21 ± 4.33 mgI/mL) was higher than that in end-inspiratory command (12.51 ± 4.38 mgI/mL) (*P *=* *.129). Generally, heavier patients are at high risk for transient interruption of contrast. Changing the inspiratory depth from end-inspiratory (9.51 ± 3.33 mgI/mL) to mid-inspiratory (11.71 ± 3.28 mgI/mL) level significantly increased the iodine concentration of the main pulmonary artery in patients with a body weight of ≥70 kg (*P *=* *.015).

Statistically significant positive correlations were found between the iodine concentration of the main pulmonary artery vs that in the right upper lobe (*r* = 0.259 [0.159–0.354], *P *<* *.001), right middle lobe (*r* = 0.241 [0.140–0.337], *P *<* *.001), right lower lobe (*r* = 0.330 [0.233–0.420], *P *<* *.001), left upper lobe (*r* = 0.274 [0.174–0.368], *P *<* *.001), and left lower lobe (*r* = 0.317 [0.220–0.408], *P *<* *.001).

### Agreement on measured iodine concentration

The intraclass correlation coefficients (1, 2) (with 95% confidence interval) of iodine concentration for right upper, right middle, right lower, left upper and left lower lobes, and main pulmonary artery were 0.790 (0.741–0.829), 0.856 (0.822–0.883), 0.807 (0.762–0.843), 0.836 (0.798–0.867), 0.836 (0.798–0.867), and 0.995 (0.994–0.996), respectively.

## Discussion

This study assessed the effect of changing the inspiratory depth on the iodine map and revealed significantly increased iodine concentrations of the right upper, right middle, and left upper lobes with the change of inspiratory depth from end- to mid-inspiratory level. The iodine concentration of the main pulmonary artery in heavier patients who are at high risk for the transient interruption of contrast was significantly increased in mid-inspiratory command compared to end-inspiratory command.

Changing the inspiratory depth from the end- to mid-inspiratory level generally reduces the incidence of transient interruption of contrast in CT pulmonary angiography.^10^ Additionally, heavier patients are at high risk for transient interruption of contrast.^11^ This study revealed increased iodine concentration of the main pulmonary artery at the mid-inspiratory level (13.21 mgI/mL) compared with that at the end-inspiratory level (12.51 mgI/mL). A significant difference in iodine concentration of the main pulmonary artery was found between these 2 inspiratory commands in patients with a body weight of ≥70 kg (*P *=* *.015).

Our study revealed that the iodine concentrations of the right upper, right middle, and left upper lobes at mid-inspiratory level (0.91, 0.74, and 0.95 mgI/mL, respectively) were significantly increased compared with those in end-inspiratory level (0.81, 0.67, and 0.85 mgI/mL, respectively) (*P *≤* *.0496). This would have been caused by the relatively higher iodine concentration of the pulmonary artery in mid-inspiratory command. A significant positive correlation between the iodine concentration of the main pulmonary artery vs that of each lobe also supports this notion. However, no statistically significant difference in iodine concentration of lower lobes was found between the 2 inspiratory depths. Pulmonary ventilation and perfusion generally match in the normal lung tissues.^12^ Additionally, the volume of the lower lobes is known to be relatively largely changed compared with that of the upper and middle lobes in the respiratory cycle.^13^ Perfusion in the lower lobes, where relatively low ventilation at the mid-inspiratory level, might have been reduced at the mid-inspiratory level, which hindered increasing iodine concentration.

Female patients have higher iodine concentrations in each lobe, and the right middle lobe demonstrated a significant difference compared to male patients. The difference in physique between males and females would not explain this phenomenon because body weight was not a significant factor for the iodine concentration of all the lung lobes. A previous study reported sex differences for myocardial perfusion.^14^ Additionally, higher myocardial perfusion was thought to be a result of higher capillary density in females. This relation may also apply to the lung; however, a detailed mechanism of the phenomenon observed in our study remains unclear.

A statistically significant difference in the iodine concentration of the lung was found between each lobe except for the right lower lobe vs the left lower lobe. Reportedly, the perfusion of the lobe is in the following relation; right lower lobe ≃ left lower lobe > right upper lobe ≃ left upper lobe > right middle lobe.^15^ Our results coincide with that previous report.

This study has some limitations. First, this study only used fast kVp switching technology while there exist some algorithms for dual-energy CT imaging. Second, the iodine concentration in lung parenchyma can be heterogeneous. However, we enhanced the reproducibility of the measured iodine concentration by averaging 2 repeated measurements. The lower limit of the 95% confidence interval for intraclass correlation coefficients (1, 2) exceeded 0.70 in all measurements. Third, there was no statistically significant difference in the iodine concentration between mid-inspiratory vs end-inspiratory for the lower zones. However, the iodine concentration in mid-inspiratory depth tended to be increased compared to that in end-inspiratory depth. Because the CT attenuation of the pulmonary artery has been reported to be increased,^10^^,^^11^ future studies investigating whether the combined use of iodine map and CT pulmonary angiography and iodine may improve the sensitivity for the detection of pulmonary embolism would be expected. Fourth, we did not use subtraction CT, which is known to show diagnostic performance comparable to that of dual-energy CT in the detection of pulmonary embolism at similar radiation dose.^16^ While we aimed to compare images acquired at end-inspiratory and mid-inspiratory levels, subtraction CT requires both the contrast-enhanced scan and unenhanced scan performed at the same inspiratory depth. Because it is rare to perform unenhanced chest CT examination at mid-inspiratory level, we did not use subtraction CT in this study. Finally, patients with lung lesions or pulmonary embolism, which make the iodine concentration measurement of parenchyma of each lobe difficult, were excluded. Our study result would not necessarily apply to such patients. It is expected that the increased iodine concentration of the healthy lung parenchyma may increase the contrast against lesion site where pulmonary embolism exists and may improve the sensitivity of iodine maps for pulmonary embolism detection. However, this topic needs to be investigated in future studies.

In conclusion, changing the inspiratory depth from end- to mid-inspiratory level helped increase the iodine concentration of the right upper, right middle, and left upper lobes. Iodine concentration of the main pulmonary artery in heavier patients who are at high risk for transient interruption of contrast was increased by changing the inspiratory depth.
